# First record of the mygalomorph spider family Paratropididae (Arachnida, Araneae) in North America with the description of a new species of *Paratropis* Simon from Mexico, and with new ultramorphological data for the family

**DOI:** 10.3897/zookeys.416.7253

**Published:** 2014-06-16

**Authors:** Alejandro Valdez-Mondragón, Jorge I. Mendoza, Oscar F. Francke

**Affiliations:** 1 Colección Nacional de Arácnidos (CNAN), Departamento de Zoología, Instituto de Biología, Universidad Nacional Autónoma de México (UNAM). 3er. Circuito exterior s/n. Apartado Postal 70-153, C.P. 04510, Ciudad Universitaria, Coyoacán, Distrito Federal, Mexico City, Mexico; 2 Alexander Koenig Research Museum of Zoology, Adenauerallee 160, 53113 Bonn, Germany.

**Keywords:** Mygalomorphae, Spider taxonomy, North America, Tropical rainforest

## Abstract

A new species of the genus *Paratropis* is described from North America: *Paratropis tuxtlensis* **sp. n.**, from a tropical rainforest in Veracruz, Mexico. This is the fifth *Paratropis* and the tenth paratropidid species described and the first North American record of this Neotropical family. The species is described based on adult males and females, and juveniles. The juveniles show ontogenetic variation in the number of cuspules on the labium and endites, and in the number and position of leg trichobothria. This is the second *Paratropis* species, and the third paratropidid known from both sexes. The scanning electron photographs (SEM) reveal new morphological data and contribute to the knowledge of the family.

## Introduction

The spider family Paratropididae Simon, 1889 is currently composed of four genera and nine species, being one of the less known spider families in the infraorder Mygalomorphae (Platnick, 2014). The genus with the highest number of species is *Paratropis* Simon, 1889 with four species: *Paratropis papilligera* F. O. P.-Cambridge, 1896, *Paratropis sanguinea* Mello-Leitão, 1923; *Paratropis scruposa* Simon, 1889; and *Paratopis seminermis* Caporiacco, 1955. The genus *Melloina* Brignoli, 1985 is composed of three species: *Melloina gracilis* (Schenkel, 1953); *Melloina rickwesti* Raven, 1999; and *Melloina santuario* Bertani, 2013. The genera *Anisaspis* Simon, 1891 and *Anisaspoides* F. O. P.-Cambridge, 1896 have one species each: *Anisaspis tuberculata* Simon, 1891 and *Anisaspoides gigantea* F. O. P.-Cambridge, 1896. Three of the nine previously described species are known only from males (*Melloina gracilis*, *Melloina santuario*, and *Paratropis papilligera*); two are known from both male and female (*Melloina santuario* and *Paratropis papilligera*); and the remaining four are known only from females. Although some species, such as the recently described *Melloina santuario* have recorded juveniles, juveniles paratropidid specimens have never been described.

The majority of the species have a natural distribution in South America, principally in Brazil, with other species distributed in Venezuela and Peru. The northern-most records of the family Paratropididae are in Central America (Panama) and the West Indies (St. Vincent) (Platnick 2014).

Raven (1999) mentioned that spiders of the family Paratropididae are enigmatic because of our limited knowledge about their habits, natural history and biology. It is known that they can be found in tropical forests, under fallen logs and boulders in the ground. As Rick West said in a personal communication to Raven (1999), these spiders make no burrow and hide under objects in the top layers of the soil. One characteristic of this family is that usually the entire body (including legs) is encrusted with soil particles (Raven 1985), although in some species such as *Melloina santuario* the cuticle has soil only in very restricted areas on the carapace (Bertani 2013). The encrusted soil on the exoskeleton could provide protection from predators or serve as camouflage to deceive their prey; it is a fact that they are very cryptic, which coupled with lack of movement when exposed makes them quite difficult to find and collect.

In this work, we describe a new species of *Paratropis* from a tropical rainforest from Veracruz, Mexico, based on juveniles and adults. It is the second species for the genus, and the third species for the family Paratropididae for which adults of both sexes are known. Juveniles specimens for the family are described for the first time. New morphological characteristics, distribution records, and natural history observations are presented.

## Materials and methods

The specimens were collected manually and deposited in ethanol (80%). Samples for future molecular studies are cold-stored in vials with ethanol (96%). The general description of the species and terminology of the chaetotaxy follows Raven (1999) and Bertani (2013) with some modifications. The description of the spinnerets follows Bertani (2013). All specimens are deposited in the Colección Nacional de Arácnidos (CNAN), Instituto de Biología, Universidad Nacional Autónoma de México (UNAM), Mexico City.

The specimens were examined, measured and photographed with a Nikon SMZ 645 stereoscope; measurements are in millimeters (mm). Female epigyna were dissected in ethanol (80%) and cleaned in KOH (10%) for 15 minutes. All structures photographed and drawn under the stereoscope were submerged first in gel alcohol (available commercially as a hand cleaner), the firm consistency of the gel allowing the immobilization and positioning of the structure. The structure suspended in the gel alcohol was then covered with liquid ethanol 80% to minimize diffraction during examination and photography. The morphological structures were dissected and cleaned (first with a needle and a fine paintbrush, then with an ultrasonic cleaner at 20–40 kHz to remove the soil particles encrusted on the exoskeleton); subsequently they were critical-point dried, and examined at low vacuum in a HITACHI S-2460N scanning electron microscope (SEM) to take the photomicrographs. All scale measurements on SEM photomicrographs are in microns. The map was done with ArcView GIS version 3.2 (Applegate 1999). The photographs and map were edited using Adobe Photoshop Version 7.0. Morphological abbreviations: ALE, anterior lateral eyes; AME, anterior median eyes; PLE, posterior lateral eyes; PME, posterior median eyes; PLS, posterior lateral spinnerets; PMS, posterior median spinnerets.

## Taxonomy

### Family Paratropididae Simon, 1889

#### Genus Paratropis

Taxon classificationAnimaliaAraneaeParatropididae

Simon, 1889

##### Type species.

*Paratropis scruposa* Simon, 1889

##### Diagnosis.

The genus can be diagnosed with the following combination of characters (after Raven 1985): 1) eye tubercle highly elevated, 2) transverse fovea, 3) narrow cheliceral furrow, with teeth on both margins in two juxtaposed rows, 4) endites with anterior conical projection, 5) Legs I of male without tibial spur, 6) paired claws of tarsus with one long tooth, 7) claw tufts absent, and 8) third claw absent on leg II (However, see Discussion concerning this character on the new species).

#### Paratropis tuxtlensis sp. n.

Taxon classificationAnimaliaAraneaeParatropididae

http://zoobank.org/8277A8CD-10F1-4ACB-B59C-0C5BB39CA60E

Figures 1 [Fig F2] [Fig F3] [Fig F4] [Fig F5] [Fig F6] [Fig F7] [Fig F8] [Fig F9] –60

##### Type material.

MEXICO: *Veracruz*: male holotype (CNAN-T0766) from Estación de Biología Tropical “Los Tuxtlas”, Universidad Nacional Autónoma de México (UNAM), Municipio San Andrés Tuxtla (18.58500°N, 95.07510°W, 1039 m), 10 November 2012; A. Valdez. O. Francke, G. Montiel, J. Cruz, R. Monjaraz Cols. Paratypes: 2 males (CNAN-T0768 and T0769), same data as holotype. 1 female (CNAN-T0822), same locality as holotype, 27 August 2005; A. Valdez. O. Francke, H. Montaño, M. Córdova, A. Jaimes Cols. 2 females (CNAN-T0767 and T0821) from 1 km SE of Díaz Ordaz, Municipio San Andrés Tuxtla (18.52775°N, 95.08691°W, 480 m), 14 June 2011; A. Valdez. O. Francke, C. Santibáñez, J. Cruz, R. Monjaraz, G. Contreras Cols.

##### Other material.

MEXICO: *Veracruz*: 1 immature (CNAN), same data as holotype. 1 immature (CNAN), same locality as holotype, 11 January 2012; O. Francke, G. Montiel, J. Cruz, R. Monjaraz Cols.

##### Diagnosis.

Distinguished from *Paratropis papilligera* (the other species where the male is known) by the male palp with conical tibia (Fig. 14), in *Paratropis papilligera* the tibia is cylindrical (F. O. P.-Cambridge 1896; fig. 7); by the pyriform palp bulb larger in *Paratropis tuxtlensis* (Figures 10–15); by the bulb with embolus shorter, almost with the same length of the palp tibia (Figure 14), and slightly sigmoid (Figures 10–15), in *Paratropis papilligera* the embolus is longer than the length of the palp tibia and more curved (F. O. P.-Cambridge 1896; fig. 7); by the number of conical teeth in the cheliceral furrows, in *Paratropis tuxtlensis* the promargin has 11 teeth and retromargin 9 (Figure 9), whereas in *Paratropis papilligera* the promargin has 14 teeth and retromargin 10.

**Figures 1–13.**
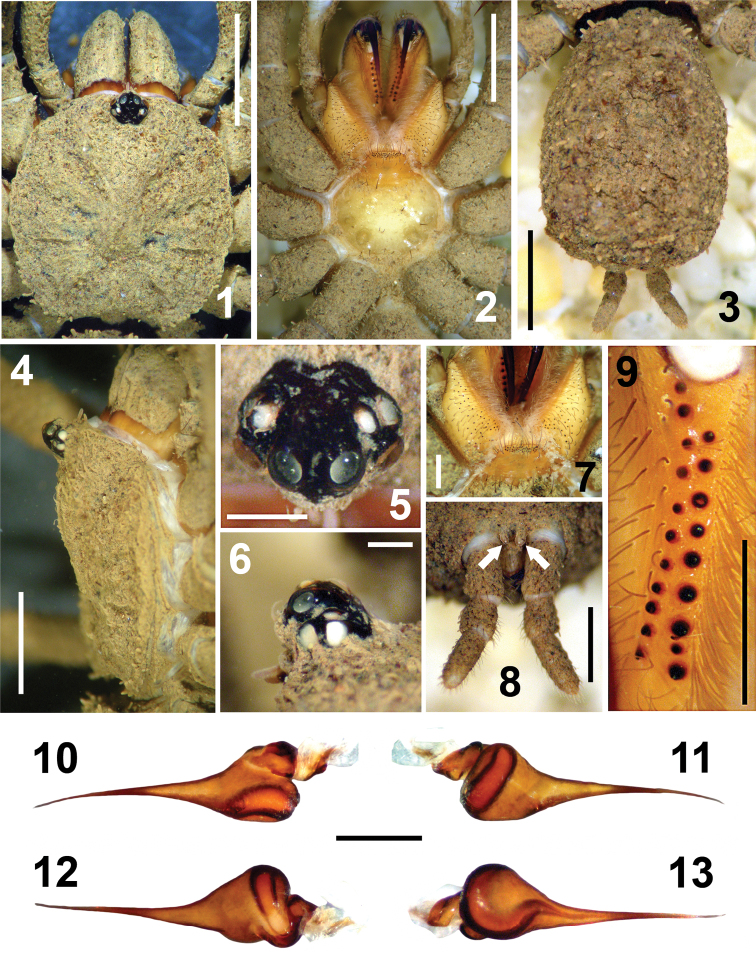
*Paratropis tuxtlensis* sp. n. Male. **1** Carapace, dorsal view **2** Prosoma, ventral view, showing the sternum, labium, endites and chelicerae **3** Opisthosoma, dorsal view **4** Carapace, right lateral view **5–6** Ocular region, dorsal and lateral views, respectively **7** Endites and labium, ventral view **8** Spinnerets, ventral view (arrows indicate the PMS) **9** Left chelicerae, teeth on promargin (left) and retromargin (right) **10–13** Bulb and embolus, prolateral, retrolateral, dorsal, and ventral views respectively. Scales: 0.4 mm (Figures **5, 6**), 0.5 mm (Figures **7, 9–13**), 1 mm (Figure **8**), 2 mm (Figures **1–4**).

**Figures 14–21. F2:**
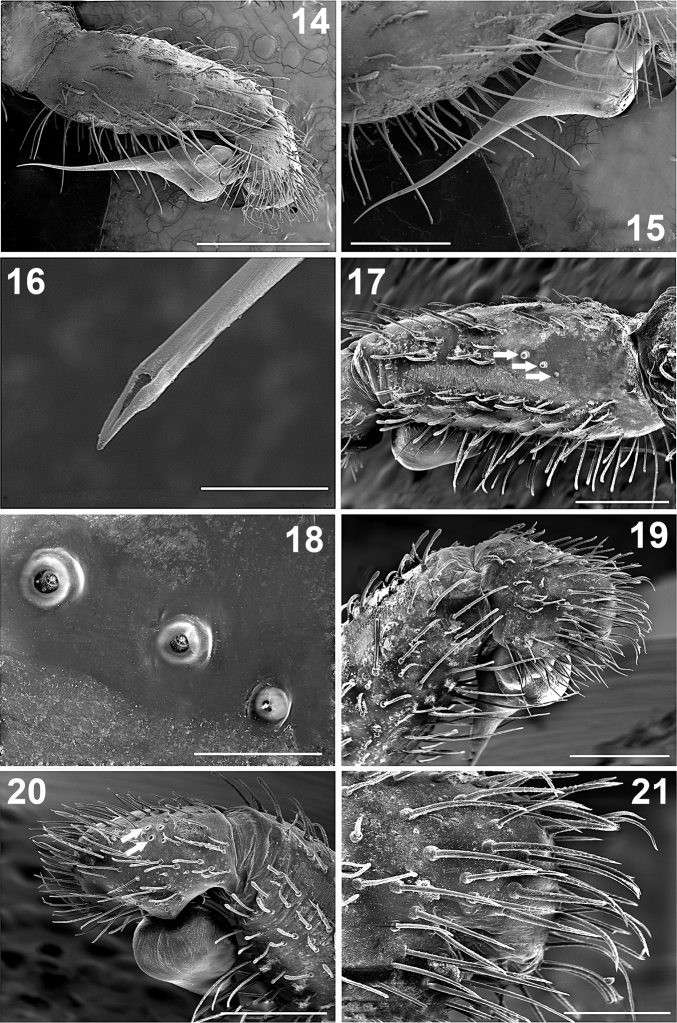
*Paratropis tuxtlensis* sp. n. Male **14** Left palp, prolateral view **15** Left palp, detail of bulb and embolus, prolateral view **16** Detail of the embolus opening, distal view **17** Left palp tibia, dorsal-retrolateral view (arrows indicate the trichobothria) **18** Detail of the trichobothria sockets on the palp tibia **19** Left palp, tibia and tarsus, prolateral view **20** Left palp, tibia and tarsus, retrolateral view (arrows indicate the trichobothria) **21** Detail of the setae on tarsus of left palp, prolateral view. Scales: 10 µm (Figure **16**), 100 µm (Figure **18**), 200 µm (Figure **21**), 500 µm (Figures **15, 17, 19, 20**), 1 mm (Figure **14**).

##### Description.

**Holotype male (CNAN-T0766).** Body length 8.20 (not including chelicerae and spinnerets); chelicerae length 1.50; carapace length 4.90, width 4.00; opisthosoma length 5.20, width 3.70.

*Coloration*: The general coloration under alcohol is the same as soil particles encrusted on the body, which is pale brown (Figures 1–4, 6, 8). Chelicerae orange ventrally (Figures 2, 7, 9), becoming brown dorsally (Figures 1, 4), fangs of chelicerae dark reddish brown (Figure 2). The carapace has reddish coloration when the soil particles are cleaned. Sternum pale orange; endites and labium orange (Figure 2). Legs olive color when soil particles are cleaned, becoming paler on tibia, metatarsi, and tarsi. The opisthosoma was difficult to clean, even with longer time in the ultrasonic cleaner, and the coloration could be similar to the carapace. Spinnerets pale yellow.

*Carapace*: Orbiculate, concave posteriorly (Figure 1). Eye tubercle elevated; fovea shallow, slightly recurved, width 0.4, visible only when the carapace is cleaned of soil encrustations. All eyes well developed; in dorsal view anterior eye row slightly recurved, posterior eye row recurved. Eye sizes and interocular distances: AME 0.24; ALE 0.26; PME 0.16; PLE 0.26; AME–AME 0.08; AME–ALE 0.04; PME–PME 0.38; PME–PLE 0.05; ALE–PLE 0.08. Ocular tubercle raised: 0.74 length; 0.88 width; clypeus lacking (Figures 5, 6).

*Palps*: Bulb pyriform (Figures 10–13, 14, 15), spermatic duct visible through integument (Figures 10–13). Embolus very long and conical, filiform apically (Figures 10–13), with spermatic opening distally (Figure 16). Tarsus with two types of setae: (i) numerous long, scattered, slightly curved, acuminate setae (Figure 21); (ii) long, clubbed setae retrolaterally (Figure 20). Tarsus with four medial-dorsal trichobothria (arrows, Figure 20). Tibiae ventrally with numerous long, curved setae (Figures 14, 15); with five trichobothria, two medial-prolateral, and three medial-retrolateral (arrows Figures 17; 18, 46). Tibiae with long, clubbed setae pro- and retrolaterally (Figures 19, 20). Patellae with numerous, curved, barbed setae. Femora concave prolaterally, dorsally with few clubbed setae on distal part. Trochanters cylindrical, with clubbed setae dorsally and ventrally. All palps segments are covered with encrusted soil particles, except the bulbs, embolus (Figures 10–13), and prolateral region of femora and trochanters.

*Chelicerae*: Cheliceral furrow promargin and retromargin with short, wide, conical teeth, wider on retromargin than on promargin (Figures 9, 30–32); promargin with 11 teeth, retromargin with 9 teeth (Figure 9); on both margins the proximal teeth are wider and longer than distal teeth (Figures 9, 31, 32). Retromargin of chelicerae with numerous long, barbed setae (Figures 30, 31), more numerous and longer than on promargin (Figure 31). Retrolateral face with clubbed setae, curved distally (Figures 36, 37), becoming shorter mesally (Figures 35, 36). Fang with venom gland duct opening dorsal sub-distal (Figures 33, 34). Cuticle on retrolateral face of chelicerae with numerous glandular pores (arrows, Figures 37).

**Figures 22–29. F3:**
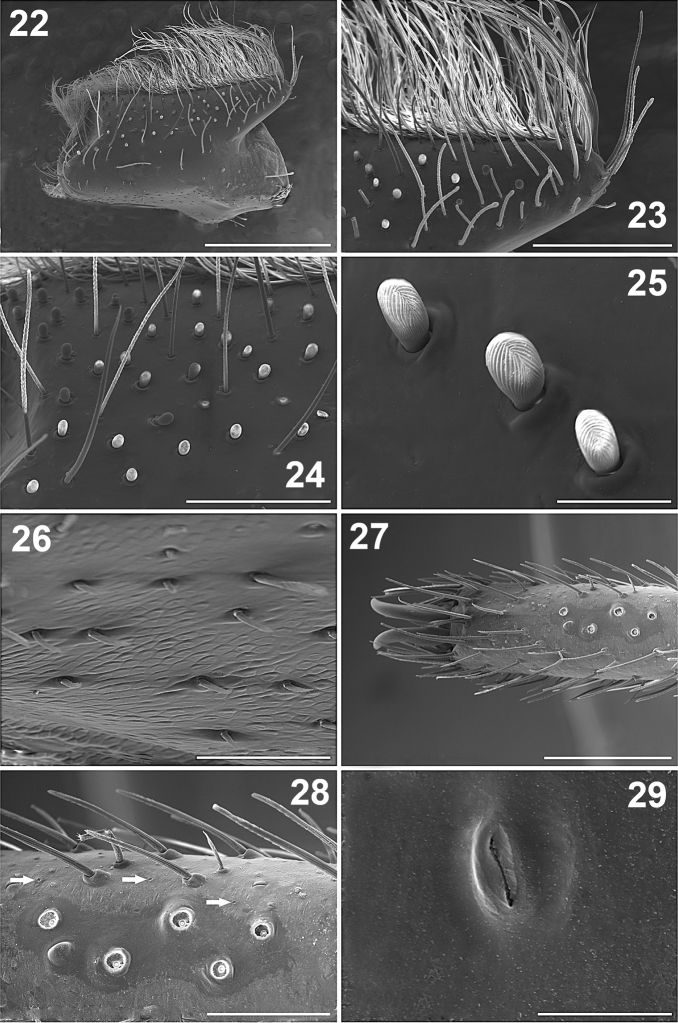
*Paratropis tuxtlensis* sp. n. Male. **22** Left endite, dorsal view **23** Left endite, apical detail **24** Left endite, detail of the setae **25** Detail of the finger-shaped cuspules on endite **26** Detail of the setae on retrolateral region of left endite **27** Left leg I, dorsal view of the tarsus **28** Left tarsus I, detail of the trichobothria sockets (arrows indicate the cuticular pores) **29** Detail of the glandular pore on the surface of the tarsus. Scales: 10 µm (Figure **29**), 50 µm (Figure **25**), 100 µm (Figure **26**), 200 µm (Figure **28**), 300 µm (Figure **24**), 400 µm (Figure **23**), 500 µm (Figure **27**), 1 mm (Figure **22**).

*Endites*: Longer than wide, with small conical projection anteriorly (Figures 2, 7, 22, 23). Prolaterally with numerous long, curved, barbed setae (Figures 22, 23), shorter proximally (Figures 22); ventrally with scattered, long, curved setae (Figures 22–24). Endites ventrally with numerous, scattered, finger-shaped cuspules; 42 cuspules on right endite and 40 on left one (Figures 7, 24, 25). Endites without pores ventrally (Figures 24, 25). Retrolateral area with small, spine-like setae (Figure 26). The cuticle is not encrusted with soil particles (Figures 2, 7).

**Figures 30–37. F4:**
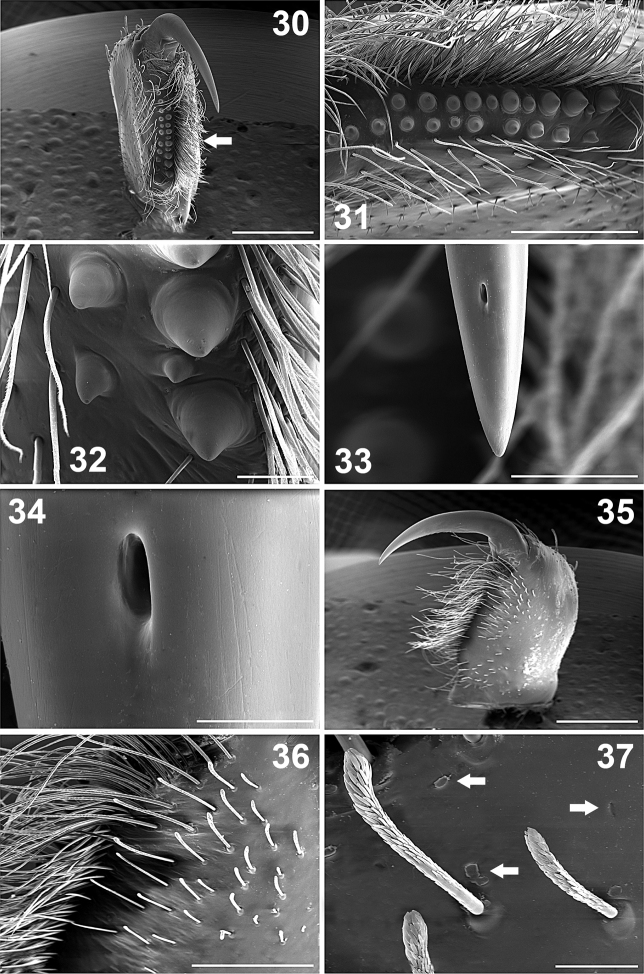
*Paratropis tuxtlensis* sp. n. Male. **30** Left chelicerae with extended fang, ventral view **31** Left chelicerae, teeth on promargin (lower line) and retromargin (upper line) **32** Detail of the chelicerae teeth **33** Left chelicerae, apical part of the fang **34** Detail of the venom gland duct opening on left fang of the chelicerae **35** Left chelicerae, retrolateral view **36** Setae on retrolateral part of left chelicerae **37** Detail of the clubbed setae on retrolateral region of chelicerae (left arrows indicate the glandular pores on the cuticle surface with secretion coming out at the moment of the microphotograph, right arrow indicates pore without secretion). Scales: 20 µm (Figure **34**), 50 µm (Figure **37**), 100 µm (Figures **32, 33**), 400 µm (Figure **36**), 500 µm (Figure **31**), 1 mm (Figures **30, 35**).

*Labium*: Trapezoidal, length 0.53, width 1.37, with 38 finger-shaped cuspules grouped on anterior part; anteriorly with several long, slightly curved setae (Figure 7); without pores on surface, not encrusted with soil particles. Labium merged to sternum, labium-sternal furrow shallow (Figures 2, 7).

*Sternum*: Circular, length 2.275, width 2.77, with few, scattered, long setae. Sigillae oval; third and fourth pairs hardly visible; fourth pair half its length from margin (Figure 2).

*Legs*: Length of legs and palp (femur, patella, tibia, metatarsus, tarsus, total): I: 5.60, 2.60, 3.75, 3.55, 1.95, 17.45. II: 3.85, 2.00, 2.70, 3.00, 1.55, 13.10. III: 3.40, 1.60, 2.25, 2.60, 1.60, 11.45. IV: 4.40, 2.00, 3.45, 3.70, 2.00, 15.55. Leg formula: 1-4-2-3. Palp: 2.00, 1.43, 1.65, -, 0.80, 5.88. Leg I longer and stouter than others, leg III shorter and thinner than others (Figure 58). Legs covered with curved, conical, barbed setae; in addition to clubbed setae (Figure 40); with numerous pores on cuticular surface (arrows, Figures 28, 40, 41), which are oval depressions with a longitudinal slit (Figure 29). Leg I without tibial spurs. Femora with long, clubbed setae. Metatarsi and tarsi with spinose setae ventrally, which are wider on legs III and IV. Tarsi with inconspicuous scopula (Figure 42), formed by small setae ending in a blunt tip (Figure 43).

**Figures 38–45. F5:**
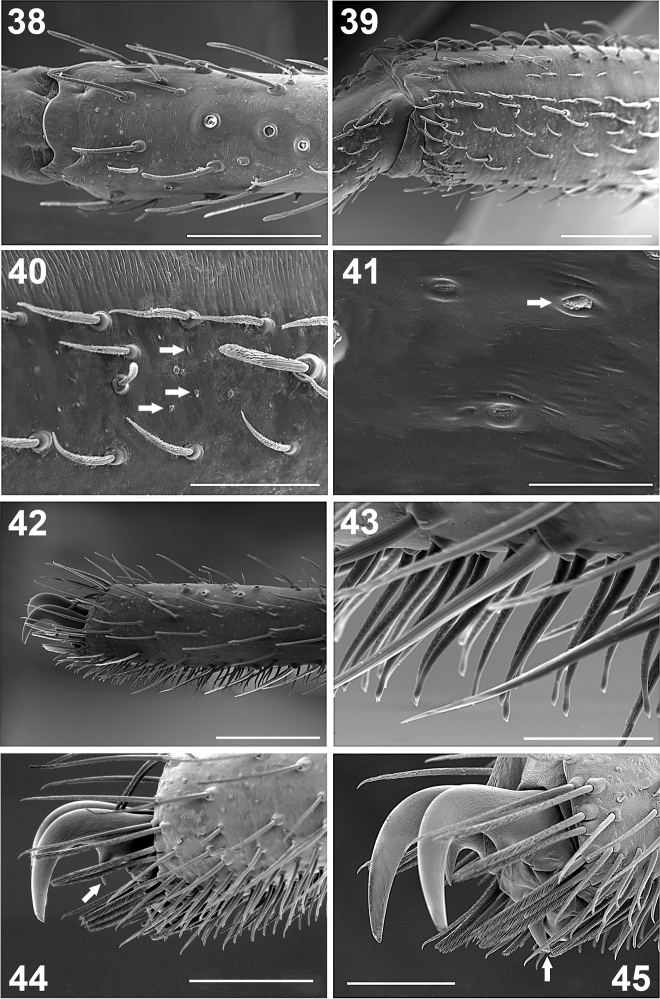
*Paratropis tuxtlensis* sp. n. Male. **38** Left leg, trichobothria of metatarsus I, dorsal view **39** Left leg, setae on tibia I, retrolateral view **40** Detail of setae on tibiae I (arrows indicate the glandular pores) **41** Detail of the glandular pores on tibia I (arrow indicates pore with secretion) **42** Left leg, tarsus I, retrolateral view **43** Detail of the scopular setae on tarsus I **44** Claws of tarsus I, retrolateral view (arrow indicates the single long tooth on left claw) **45** Detail of the paired claws of tarsus I (arrow indicates the unpaired ventral claw). Scales: 40 µm (Figure **41**), 100 µm (Figure **43**), 200 µm (Figures **40, 45**), 300 µm (Figure **44**), 400 µm (Figure **38**), 500 µm (Figures **39, 42**).

*Claws*: Tarsi with long paired claws (Figures 42, 44, 45), which have just one long median tooth ventrally (arrow, Figure 44). Only tarsus I has the third, unpaired claw (arrow, Figure 45), tarsus I ventral-distally with some barbed setae, near to unpaired claw (Figure 45).

**Figures 46–47. F6:**
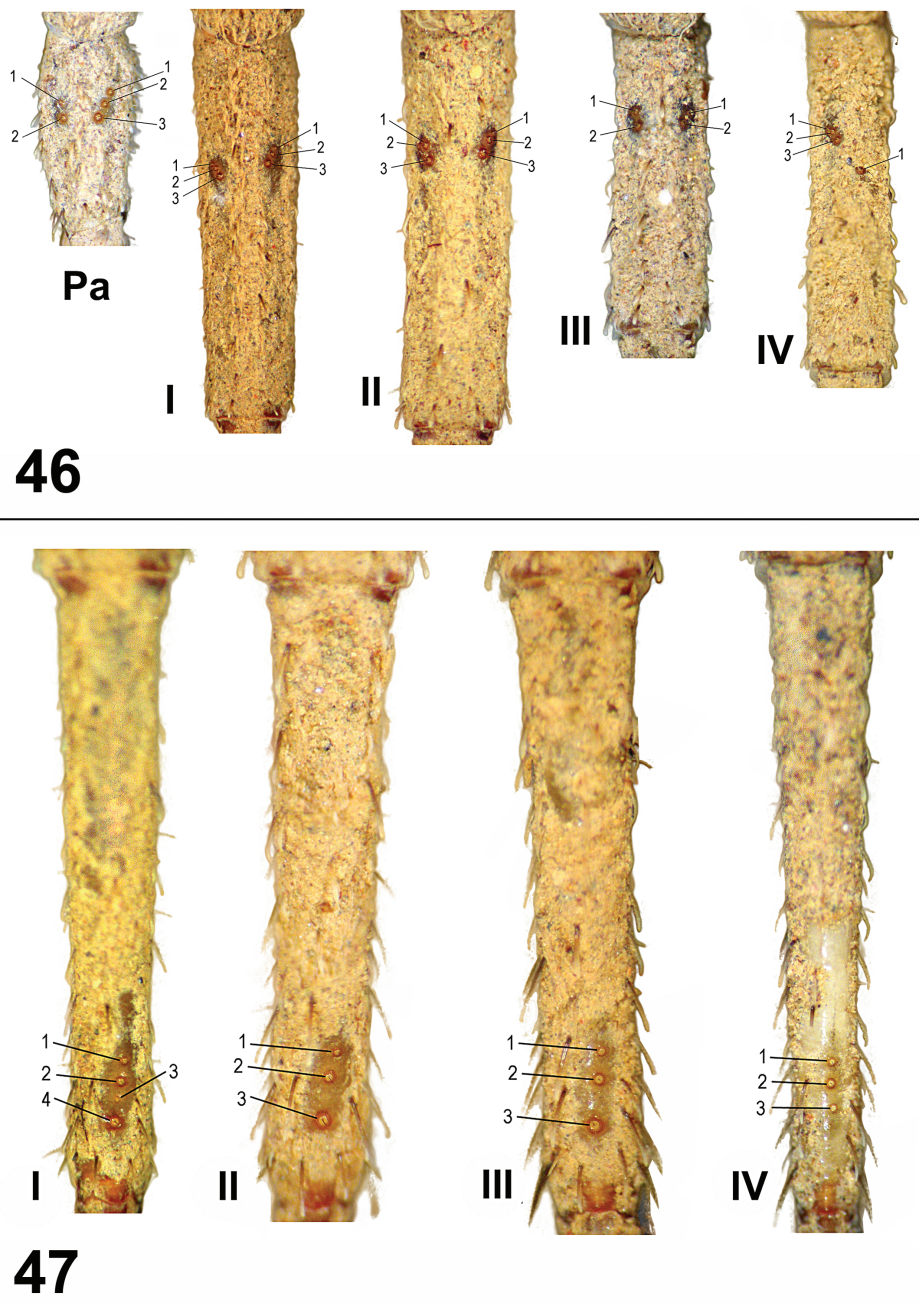
*Paratropis tuxtlensis* sp. n. Trichobothria on male appendages **46** Trichobothria pattern on tibiae of palp (Pa) and legs I-IV **47** Trichobothria pattern on metatarsi I-IV.

**Figures 48. F7:**
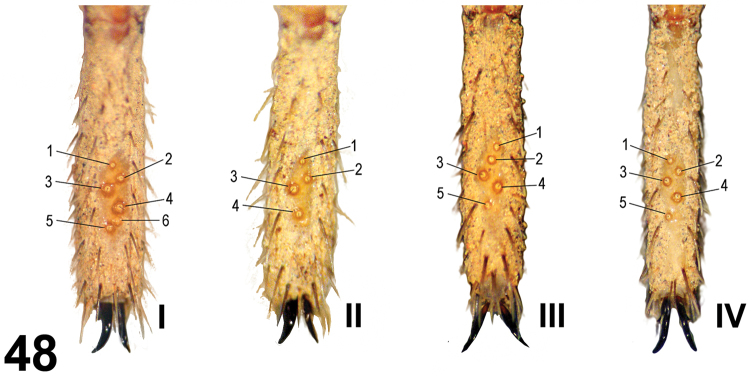
*Paratropis tuxtlensis* sp. n. Trichobothria pattern on tarsi I-IV on male legs.

*Leg trichobothria*: Located on tibiae, metatarsi, and tarsi (Figures 46–48). Cuticle around the trichobothria without soil-particle encrustations (Figures 18, 28, 38, 46–48). Trichobothria sockets variable in size, basal-most smallest (Figures 18, 28, 38, 46–48). Dividing each leg segment into thirds (basal, median, apical), tibiae I has six trichobothria, three medial-prolateral, three medial-retrolateral (Figure 46). Tibia II has six trichobothria, three medial-prolateral, three medial-retrolateral (Figure 46). Tibia III has four trichobothria, two medial-prolateral, two medial-retrolateral (Figure 46). Tibia IV has four trichobothria, three medial-prolateral, one medial-retrolateral (Figure 46). All metatarsi have three trichobothria apical-dorsal (Figure 47). Finally, tarsus I has six trichobothria medial-dorsal, tarsus II has four medial-dorsal, tarsi III and IV have five trichobothria medial-dorsal (Figure 48).

*Chaetotaxy (left side)*: Metatarsus III 1v; IV 1v. The legs on males have numerous conical, barbed setae.

*Opisthosoma*: Oval, longer than wide (Figure 3), dorsally with eight longitudinal rows of clubbed setae, each row with eight setae. Opisthosoma completely covered with soil particles (Figure 3), genital gonopore not visible. Booklung openings oval, sclerotized.

*Spinnerets*: PMS considerably shorter than PLS (arrows Figure 8). First and second segments of PLS cylindrical, third segment finger-shaped distally (Figure 8). Measurements: PMS length 0.22, width 0.12, 0.10 apart. Segments of PLS (length): basal 0.70, middle 0.50, distal 0.90; midwidths PLS (width): basal 0.48, middle 0.46, distal 0.34.

**Paratype female (CNAN-T0767).** Body length 12.90 (not including chelicerae and spinnerets); chelicerae length 1.80; carapace length 6.00; width 5.70; opistosoma length 6.40, width 5.10.

**Female similar to the male, differences:** *Coloration*: Chelicerae, endites, labium, and sternum darker orange than the male (Figures 50, 55).

**Figures 49–56. F8:**
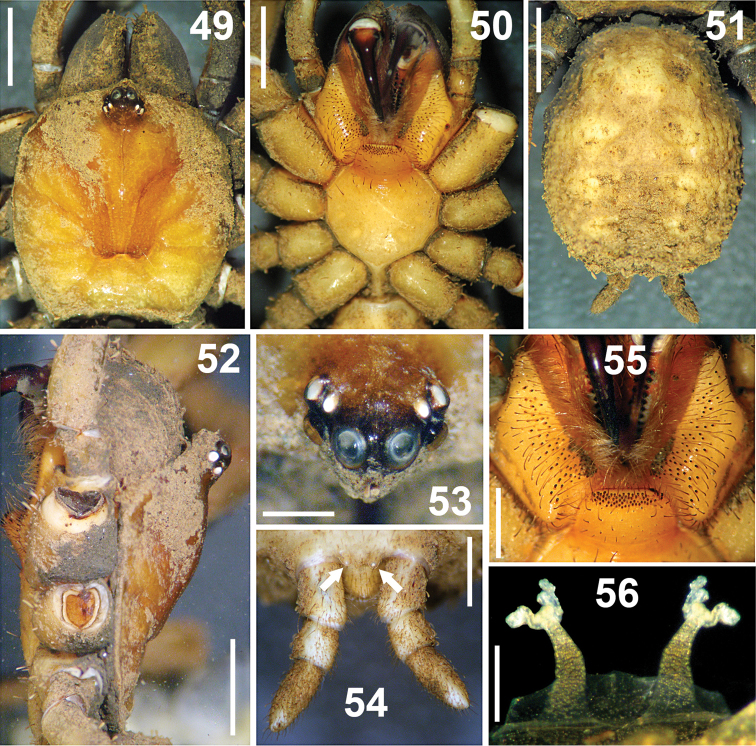
*Paratropis tuxtlensis* sp. n. Female. **49** Carapace, dorsal view **50** Prosoma, ventral view, showing the sternum, labium, endites and chelicerae **51** Opisthosoma, dorsal view **52** Carapace, left lateral view **53** Ocular region, dorsal view **54** Spinnerets, ventral view (arrows indicate the PMS) **55** Endites and labium, ventral view **56** Spermathecae, dorsal view. Scales: 0.25 mm (Figure **56**), 0.6 mm (Figure **53**), 1 mm (Figures **54, 55**), 2 mm (Figures **49–52**).

*Carapace*: Oblong-orbiculate (Figure 49). Caput elevated (Figure 52); fovea shallow, slightly recurved, 1.08 wide, visible only when carapace is cleaned of soil particle encrustations. Eye sizes and interocular distances: AME 0.35; ALE 0.25; PME 0.15; PLE 0.28; AME–AME 0.13; AME–ALE 0.05; PME–PME 0.55; PME–PLE 0.06; ALE–PLE 0.08. Ocular tubercle raised; length 1.08, width 1.13; clypeus lacking (Figure 53).

*Palps*: Thicker than on male. Tarsi with one distal, long, curved unpaired claw, which has just one tooth. Tarsi ventrally with spines; left tarsus with spines: 2+1, right tarsus with spines: 1+2+2. All palp segments covered with encrusted soil particles, except prolateral regions of femora and trochanters. Tibia with five trichobothria, two medial-prolateral, three medial-retrolateral. Tarsus with four medial-dorsal trichobothria.

*Chelicerae*: Fangs wider than on male (Figure 50). Chelicerae furrows with conical, wide, short teeth on promargin and retromargin. Right chelicera promargin with 13 teeth, retromargin with 11 teeth; left chelicera promargin with 12 teeth, retromargin with 11 teeth; teeth on retromargin wider than teeth on promargin; on both rows the proximal teeth are wider and longer than distal teeth.

*Endites*: Endites ventrally with numerous scattered, finger-shaped cuspules, 61 cuspules on right endite and 64 on left one (Figure 55).

*Labium*: Trapezoidal, length 1.02, width 1.58, with 69 cuspules grouped on anterior region; with several long and slightly curved setae on anterior part. Cuspules finger-shaped, as on endites. Labium merged to sternum, without glandular pores on surface, cuticle not encrusted with soil. Labium-sternal furrow shallow (Figure 55).

*Sternum*: Circular, length 2.5, width 3.05; with few scattered, long setae. Sigillae oval, third and fourth pairs hardly visible; fourth pair 3/4 times its length from margin.

*Legs*: Length of legs and palp (femur, patella, tibia, metatarsus, tarsus, total): I: 3.65, 2.65, 3.25, 2.70, 1.40, 13.65. II: 3.50, 2.05, 2.35, 2.50, 1.45, 11.85. III: 2.95, 1.85, 2.05, 2.30, 1.35, 10.50. IV: 4.00, 1.60, 3.10, 3.40, 1.75, 13.85. Leg formula: 4-1-2-3. Palp: 2.67, 1.67, 1.53, -, 1.90, 7.77. Metatarsi and tarsi with spinose setae ventrally, wider on legs III and IV; thicker and more visible than on male. Tarsi with inconspicuous scopula, composed of small setae ending in blunt tip.

*Claws*: Slightly longer than the male. Only the tarsi I and II with small, unpaired third claw (differing from the male, which lacks it in tarsus II, and from Raven’s 1985 generic diagnosis). Palp tarsus with one single claw, without tooth.

*Leg trichobothria*: Located on tibiae, metatarsi, and tarsi. Cuticle around trichobothria not covered with encrusted soil particles. Trichobothrial socket size variable, smallest basally and apically. Tibia I has six trichobothria: three medial-prolateral, two medial-retrolateral, one medial-dorsal; tibia II has six trichobothria: three medial-prolateral, three medial-retrolateral; tibia III has six trichobothria: three medial-prolateral, three medial-retrolateral; tibia IV has five trichobothria: one medial-prolateral, one medial-dorsal, three medial-retrolateral; palpal tibia has six trichobothria: three medial-prolateral, three medial-retrolateral. Metatarsi I-III has three apical-dorsal trichobothria, metatarsus IV has four trichobothria: one medial-dorsal, three apical-dorsal. Tarsus I has nine medial-dorsal trichobothria; tarsus II has seven medial-dorsal trichobothria; tarsus III has six medial-dorsal trichobothria; tarsus IV has seven medial-dorsal trichobothria. Palpal tarsus has five medial dorsal trichobothria.

*Chaetotaxy (left side)*: Metatarsus I 28v; II 6v; III 5v; IV 3v; tarsus I 14v; II 7v; III 3v; IV 1v; palp 7v. Conspicuous spines are more visible in females than in males.

*Opisthosoma*: Bigger than in male (Figure 51), genital operculum not visible due to encrusted soil particles.

*Spermathecae*: Two long, separated lobes, wider basally, slightly curved outwards from base; apically with paired sigmoid receptacles (Figure 56).

*Spinnerets*: PMS considerably shorter than PLS (arrows Figure 54), however PLS bigger than on male (Figure 54). First and second segments of PLS cylindrical, third segment finger-shaped distally. Measurements: PMS length 0.24, width 0.18, 0.24 apart; Segments of PLS (length): basal 0.90, middle 0.46, distal 1.00; midwidths PLS (width): basal 0.64, middle 0.58, distal 0.46 (Figure 54).

##### Variation.

Males (N= 3), females (N= 3). There is no variation in male secondary sexual characteristics. It is difficult to determine if there is variation in coloration in males due to the soil particles encrusted on the cuticle. However, in the endites, labium, and sternum which do not have encrusted soil particles, there was no variation in coloration. *Males*: Carapace length 4.50–5.35 (*x* = 4.88), width 4.7–5.2 (*x* = 4.93). Tibia I length 3.90–4.50 (*x* = 4.25). Sternum length 2.25–2.50 (*x* = 2.35), width 2.55–2.85 (*x* = 2.70). Endites length 2.00–2.30 (*x* = 2.13). Cuspules: endites, male 1 (right/left) (42/40), male 2 (38/39), male 3 (54/48); labium, male 1 (39), male 2 (47), male 3 (32). *Females*: Carapace length 5.40–6.10 (*x* = 5.80), width 5.10–5.80 (*x* = 5.50). Tibia I length 3.65–4.00 (*x* = 3.81). Sternum length 2.40–2.85 (*x* = 2.68), width 3.00–3.30 (*x* = 3.16). Endites length 2.45–2.75 (*x* = 2.61). Cuspules: endites, female 1 (60/66), female 2 (75/77), female 3 (43/51); labium, female 1 (64), female 2 (68), female 3 (41).

Juveniles (N= 2) (two different instars): Body lengths 4.30 (#1), 5.10 (#2) (not including chelicerae and spinnerets); Carapace lengths 2.05, 2.25, widths 1.95, 2.37. Tibia I lengths 1.35, 1.55. Sternum lengths 1.10, 1.30, widths 1.40, 1.55. Endites lengths 0.86, 1.00. Cuspules: endites, juvenile #1 (right/left) (12/12), juvenile #2 (19/17); labium: juvenile 1 (14), juvenile 2 (15). *Leg trichobothria*: Juvenile #1: Tibia I (4 trichobothria) (2 medial-prolateral, 2 medial-retrolateral), tibia II (4) (2 medial-prolateral, 2 medial-retrolateral), tibia III (4) (2 medial-prolateral, 2 medial-retrolateral), tibia IV (3) (2 medial-prolateral, 1 medial-retrolateral). Metatarsus I (2) (1 medial-dorsal, 1 apical-dorsal), metatarsus II (2) (1 medial-dorsal, 1 apical-dorsal), metatarsus III (2) (1 medial-dorsal, 1 apical-dorsal), metatarsus IV (2) (apical-dorsal). All tarsi (2) (medial-dorsal). Juvenile #2: Tibia I (4) (2 medial-prolateral, 2 medial-retrolateral), tibia II (4) (2 medial-prolateral, 2 medial-retrolateral), tibia III (4) (2 medial-prolateral, 2 medial-retrolateral), tibia IV (3) (2 medial-prolateral, 1 medial-retrolateral). All metatarsus (2) (apical-dorsal). Tarsus I (4) (medial-dorsal), tarsi II-IV (3) (medial-dorsal). *Palp trichobothria*: Juvenile #1: Tibiae (3) (1 medial-prolateral, 2 medial-retrolateral), Tarsus (2) (medial-dorsal). Juvenile #2: Tibiae (3) (1 medial-prolateral, 2 medial-retrolateral), Tarsus (2) (medial-dorsal).

##### Etymology.

The specific name is an adjective and refers to the type locality: Estación de Biología Tropical “*Los Tuxtlas*”, Municipio San Andrés Tuxtla, Veracruz, Mexico.

##### Distribution.

The species is known only from the region around the type locality in the Volcan San Martin Biosphere Reserve (Figure 62).

##### Natural history.

**Figure 57. F9:**
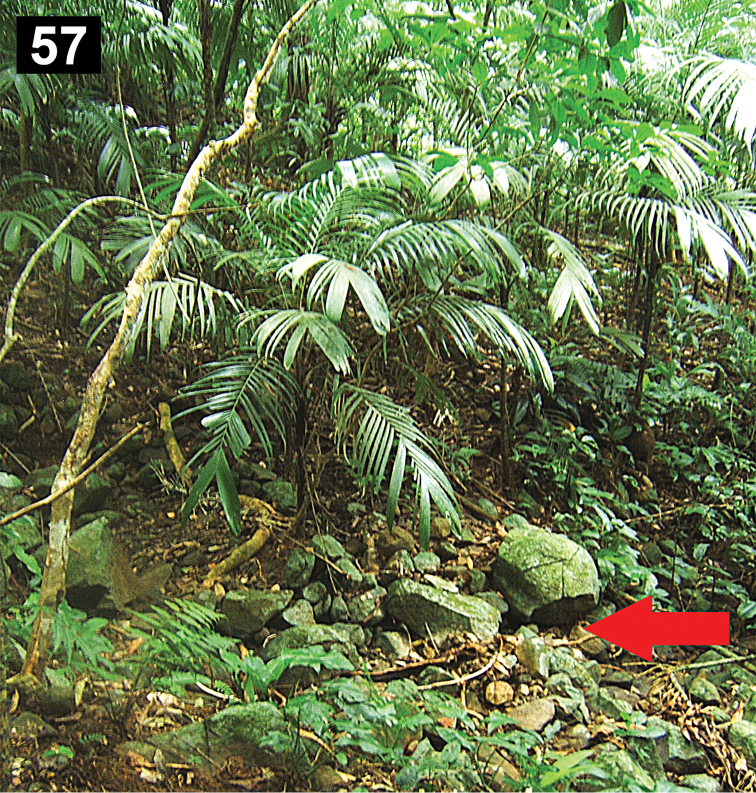
Tropical rain-forest in the Estación de Biología Tropical “Los Tuxtlas”, Veracruz, Mexico. Habitat of *Paratropis tuxtlensis* new species, arrow indicates the microhabitat where the specimens were collected (under boulders).

**Figures 58–61.**
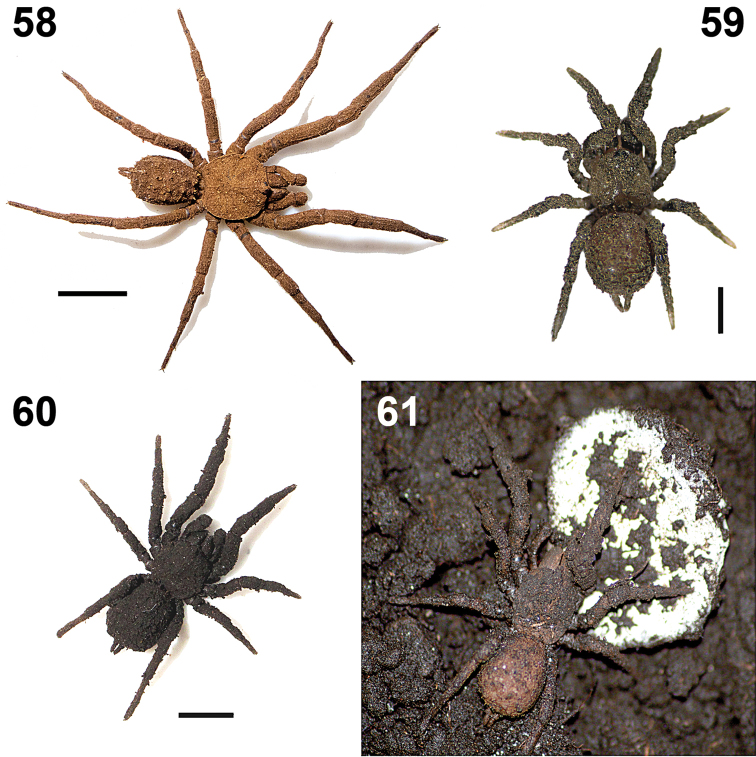
*Paratropis tuxtlensis* sp. n. Photographs of live specimens, kept in the laboratory **58** Adult male **59** Immature specimen **60** Adult female **61** Adult female protecting her egg sac. Scales: 1 mm (Figure **59**), 4 mm (Figure **58**), 6 mm (Figure **60**).

The specimens were collected in tropical rain-forest, under boulders on the ground (Figure 57). The holotype, two paratype males and one juvenile where collected near each other, within around 3 m^2^, in a zone with numerous small and big boulders on the ground. The specimens remained motionless when they were exposed by removing the rock that provided shelter, possibly as a defense mechanism because the soil particles encrusted on the body cuticle serves as camouflage with the moist ground (Figures 57–60). The type locality is at 1039 m elevation, and two adult females where collected nearby at 480 m.

**Figure 62.**
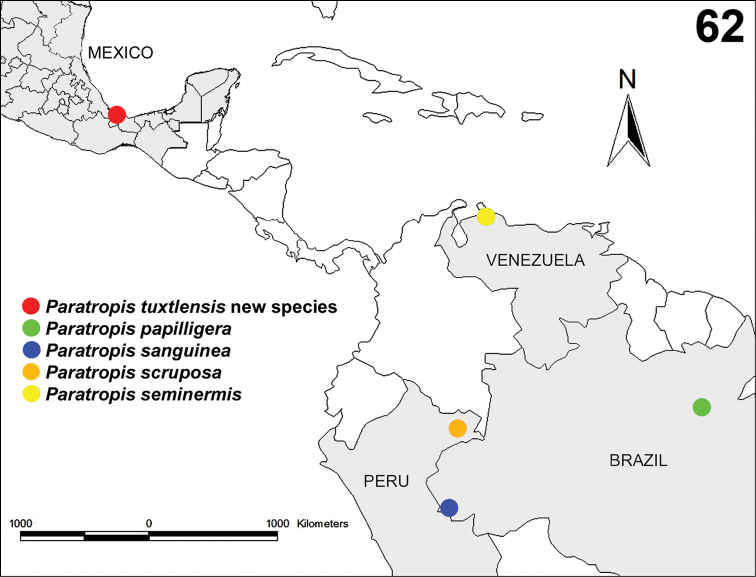
Known records of the species of the genus *Paratropis*: *Paratropis tuxtlensis* new species from Estación de Biología Tropical “Los Tuxtlas”, Veracruz, Mexico; *Paratropis papilligera* from Santarém, Pará, Brazil; *Paratropis sanguinea* from Alto-Jurúa, Amazonas, Brazil; *Paratropis scruposa* from Pebas, Loreto, Peru; and *Paratopis seminermis* from Santa Ana, Falcón, Venezuela.

Towards the end of spring (May 19, 2012), one paratypes female (CNAN-T0767) kept in captivity in the laboratory laid an egg sac (Figure 61). The female kept her palps and legs in contact with the egg sac constantly. Twenty-three spiderlings emerged 38 days after oviposition (July 26, 2012).

##### Remarks.

Although *Paratropis papilligera* was described by F. O. P.-Cambridge (1896) based on male and female adults from Pará, Brasil (F. O. P.-Cambridge 1896; figs 1, 6-8, 17, 23), being the first species where the male is known, the type material of *Paratropis papilligera* for the comparative description of *Paratropis tuxtlensis* sp. n. was not revised because the description made by F. O. P.-Cambridge (1896) is complete and enough. The detailed F. O. P.-Cambridge’s description allowed getting enough information about the important morphological characters to separate *Paratropis tuxtlensis* as a new species as was mentioned in the comparative diagnosis herein.

## Discussion

The spider family Paratropididae Simon, 1889 was considered as being monophyletic and as the sister group of the family Theraphosidae Thorell, 1869 (Superfamily Theraphosoidea) based on morphological evidence by Raven (1985) and Goloboff (1993). However, molecular data are in conflict with morphological evidence (Hedin and Bond 2006; Bond et al. 2012). This enigmatic family appears to be difficult to place phylogenetically, due to the available material for molecular phylogenetic analyses being inadequate to evaluate the monophyly of the family. The last phylogenetic analysis made by Bond et al. (2012) using total evidence, found to Paratropididae as the sister group of the clade Bipectina (Goloboff 1993) as was found by Hedin and Bond (2006).

The monophyly of the four genera that compose the family has never been tested. Even the monophyly of the subfamilies proposed by Simon (1889) (Paratropidinae), and Raven (1985) (Glabropelmatinae) have never been tested; although characters as the single tooth on the claws, the steeply elevated eye tubercle, the absence of a tibial spur on leg I, and the lack of claw tufts have been useful to diagnose Paratropidinae; whereas in the case of Glabropelmatinae the presence of claw tufts, a normally elevated eye tubercle, and shorter anterior maxillary lobes are diagnostic.

We report the ontogenetic variation for Paratropididae juveniles for the first time. The number of the cuspules on labium and sternum is considerably lower in younger instars than on adults. We found two juveniles that we hypothesize belong to two different, not necessarily successive instars; the smallest had fewer cuspules on labium and endites than the larger juvenile and adults. It seems that the number of cuspules in each instar is incremented with growth, and the adult instars have the highest number. In addition, there is ontogenetic variation in the number of leg trichobothria in each instar, with fewest trichobothria on the younger instars and the highest number on the adults. Although we hypothesize that the juveniles belong to two different, not successive instars, based on size differences, both specimens had the same number of trichobothria on tibiae and metatarsi. However, there is allometric growth reflected in the trichobothria position on metatarsi: in the smallest juvenile one trichobothrium is located on the median third and the other one in the apical third; whereas in the larger juvenile both trichobothria are located in the apical third. Although on both juvenile specimens the trichobothria number on tibiae and metatarsi was the same, the number of trichobothria on the tarsi is different in the larger juvenile, having one more trichobothrium.

The genus *Paratropis* Simon, 1889 was diagnosed by Simon (1889) based only on the combination of somatic characters, some of them shared with other genera in the family. The diagnosis was based on the shape of the ocular tubercle, size of eyes, shape of the endites and labium, presence of scopules on labium, leg formula, and the presence of clubbed setae along the opisthosoma. However, Simon did not describe any sexual characters; although *Paratropis scruposa* (type species) was described based on one female, he did not describe the spermathecae, which have been traditionally useful not only at level species but also at generic level in the taxonomy of spiders. Although Raven (1985) did not diagnose the genus, he mentioned the combination of some somatic characters, as Simon had done, that could be used to characterize it. In the key to genera he mentioned that *Paratropis* can be identified by the third, unpaired claw absent on leg II (Raven 1985; page 122). However, the female of the new species described here has tarsi I and II with a small, unpaired claw, which could call into question the validity of that diagnostic character for the genus, or could lead to the recognition of a new genus in the family Paratropididae. Because to the discrepancies mentioned above, robust cladistic analyses based on morphological and molecular data are necessary to test the validity of *Paratropis* and the other genera in the family. It is urgent to collect the males of most of the species, because *Paratropis tuxtlensis* is only the second species of the genus, and the fourth species of the family, where the male is known. The structures of male palps might provide additional information not only related with the diagnoses at species level, but also potential phylogenetic information within the genus and the relationships of the genera of Paratropididae.

## Supplementary Material

XML Treatment for Paratropis

XML Treatment for Paratropis tuxtlensis

## References

[B1] Applegate AD (1999) ArcView GIS version 3.2. Environmental Systems Research Institute, Inc. Neuron Data, Inc.

[B2] Bertani R (2013) A new species of *Melloina* (Araneae: Paratropididae) from Venezuela. Zoologia 30(1): 101–106. doi: 10.1590/S1984-46702013000100013

[B3] Cambridge FOP (1896) On the Theraphosidae of the lower Amazons: being an account of the new genera and species of this group of spiders discovered during the expedition of the steamship "Faraday" up the river Amazons. Proceedings of the Zoological Society of London, 716–766

[B4] Goloboff PA (1993) A reanalysis of mygalomorph spider families (Araneae). American Museum Novitates 3056: 1–32

[B5] Hedin M Bond JE (2006) Molecular phylogenetics of spider infraorder Mygalomorphe using nuclear rRNA genes (18S and 28S): Conflict and agreement with the current system of classification. Molecular Phylogenetics and Evolution 41: 454–471. doi: 10.1016/j.ympev.2006.05.017 16815045 10.1016/j.ympev.2006.05.017

[B6] Bond JE Hendrixon BE Hamilton CA Hedin M (2012) A Reconsideration of the Classification of the Spider Infraorder Mygalomorphae (Arachnida: Araneae) Based on Three Nuclear Genes and Morphology. PLoS ONE 7: . doi: 10.1371/journal.pone.0038753 10.1371/journal.pone.0038753 PMC3378619 22723885

[B7] Platnick NI (2014) The World Spider Catalog, version 14.5. American Museum of Natural History. doi: 10.5531/db.iz.0001

[B8] Raven RJ (1985) The spider infraorder Mygalomorphae (Araneae): cladistics and systematics. Bulletin of the American Museum of Natural History 182: 1–180

[B9] Raven RJ (1999) Review of the mygalomorph genus *Melloina* Brignoli (Paratropididae: Araneae). Memoirs of the Queensland Museum 43(2): 819–825

[B10] Simon E (1889) Voyage de M. E. Simon au Venezuela (décembre 1887-avril 1888). 4o. mémoire. Arachnides. Ibid., ser. 6, vol. 9, 169–220, pls. 1–3.

